# Selectively reduced contrast sensitivity in high schizotypy

**DOI:** 10.1007/s00221-019-05695-9

**Published:** 2019-11-28

**Authors:** Lauren Harper, Emily Spencer, Colin Davidson, Claire V. Hutchinson

**Affiliations:** 1 Department of Neuroscience, Psychology and Behaviour, College of Life Sciences, University of Leicester, Leicester, UK; 2 School of Pharmacy and Biomedical Sciences, Faculty of Clinical and Biomedical Sciences, University of Central Lancashire, Preston, UK

**Keywords:** Vision, Spatial frequency, Temporal frequency, Schizotypy, Psychosis proneness

## Abstract

Deficits in the ability to encode small differences in contrast between adjacent parts of an image (contrast sensitivity) are well documented in schizophrenic patients. In the present study, we sought to determine whether contrast sensitivity deficits reported in schizophrenic patients are also evident in those who exhibit high schizotypy scores in a typical (i.e., non-schizophrenic) population. Using the O-Life Questionnaire, we determined the effects of schizotypy on spatial (0.5, 2 and 8 c/deg) and spatiotemporal (0.5 and 8 c/deg at 0.5 and 8 Hz) contrast sensitivity in 73 young (18–26 years), majority female (*n* = 68) participants. We found differences in contrast sensitivity that were spatial, spatiotemporal and O-Life subscale specific. Spatial contrast sensitivity was significantly lower in high, compared to low schizotypes at low spatial frequencies (0.5 c/deg) in those who scored highly on the Unusual Experiences and Cognitive Disorganisation O-Life subscales. For moving stimuli, individuals with high scores on the Unusual Experiences subscale exhibited lower spatiotemporal contrast sensitivity for 0.5 and 8 c/deg patterns drifting at 8 Hz. Although the effects reported here were relatively small, this is the first report of reduced contrast sensitivity in schizotypy.

## Introduction

It has long been known that dopamine is involved in multiple visual processes (Djamgoz et al.^1997^) including contrast sensitivity (Tagliati et al. 1994). This has been shown in both animal (Bodis-Wollner 1990) and human studies (Masson et al. 1993). This should be no surprise as dopamine is found in abundance in the retina (Brandies and Yehuda 2008) and dopamine neurons innervate visual cortex (Jacob and Nienborg 2018). Visual problems are characteristic of neurodegenerative diseases known to affect the dopamine system. Parkinson’s patients, for example, whose cardinal neuropathology is degeneration of the dopamine system, exhibit reduced contrast sensitivity, even in the early stages of Parkinson’s Disease (Ming et al. 2016). Furthermore, contrast sensitivity deficits appear to be associated with cognitive deficits in this patent group (Ridder et al. 2017).

Schizophrenia represents another example of a well-known brain disorder characterised by a dysfunctional dopamine system (Howes et al. 2017), although it may also involve dysfunction in serotonin, GABA and glutamate systems (Yang and Tsai 2017). Dysfunctional visual attention, cognition and executive processing are well documented in schizophrenia (Harvey et al. 2001; Barch and Ceaser 2012). Deficits in low-level visual perceptual processing have also been reported, and it has been suggested that tests of low-level vision should be included in diagnostic test batteries (Butler et al. 2008). The most widely studied low-level visual deficit in schizophrenia concerns the ability of schizophrenics to encode stimulus contrast. Reduced contrast sensitivity can be present even when there is no detectable impairment in visual acuity. Such reductions provide a sensitive clinical measure of visual function and can reveal abnormal visual processing at the level of the retina and in the cortical and subcortical visual pathways (Owsley 2003). Schizophrenics require significantly more contrast between adjacent parts of an image to detect that they are different.

A number of studies have assessed conventional psychophysical contrast sensitivity (i.e., thresholds for determining the presence of a luminance-defined sinusoidal grating) in schizophrenia. The majority, but not all, studies find some evidence of schizophrenia-related contrast sensitivity deficits (Slaghuis 1998, 2004; Chen et al. 1999, 2003; Keri et al. 2002; Butler et al. 2005, 2009; Cimmer et al. 2006; Calderone et al. 2013; Cadenhead et al. 2013; Shoshina et al. 2014; Samani et al. 2018). However, in studies where deficits are reported, there is variation in the spatial and temporal frequency specificity of deficits between different patient groups and across studies. As such, at present, the clinical utility of contrast sensitivity testing in schizophrenia is somewhat limited. A summary of key studies and their findings is provided in Table 1.

**Table 1:** Summary of the effects of schizophrenia on conventional contrast sensitivity for stationary and moving grating patterns

Study	Groups	Spatial and temporal frequencies tested	Key findings
Slaghuis (1998)	30 schizophrenic patients, 15 controls	1 and 8 c/deg	Reduced CS in schizophrenic patients at both spatial frequencies compared to controls
Chen et al. (1999)	15 schizophrenic patients, 18 controls	0.5 c/deg; 0, 5 Hz	No difference in CS between patients and controls
Keri et al. (2002)	20 schizophrenic patients, 15 controls	0.5, 1.2, 1.9, 2.9, 3.6, 4.8, 5.7, 7.2 and 14.4 c/deg, 0, 8 Hz	Reduced CS in schizophrenic patients across all conditions compared to controls For stationary patterns, deficits were most pronounced at spatial frequencies above 2 c/deg
Chen et al. (2003)	45 schizophrenic patients: 8 prescribed typical antipsychotics, 25 prescribed atypical antipsychotics, 6 prescribed combination typical and atypical antipsychotics, 8 not prescribed antipsychotics–although 3 of these were taking SSRIs, 24 first degree relatives, 39 controls	0.5 c/deg, 0.5 Hz	Reduced CS in the schizophrenic group compared to controls Reduced CS in patients prescribed typical antipsychotics than patients prescribed atypical antipsychotics and controls Better CS in patients not prescribed antipsychotics compared to controls No differences between: (i) patients prescribed atypical antipsychotics and controls; (ii) patients prescribed a combination of typical and atypical antipsychotics and those prescribed only atypical antipsychotics, only typical antipsychotics, or controls; (iii) first degree relatives and controls
Slaghuis (2004)	28 schizophrenic patients (14 positive symptom patients, 14 negative symptom patients), 14 controls	0.5, 2, 4, 8 c/deg; 0, 4, 8, 16 Hz	Reduced CS in schizophrenic patients but only those assigned to the ‘negative symptom’ group
Butler et al. (2005)	33 schizophrenic patients, 21 controls	0.5, 1, 2, 4, 7, 10, 21 c/deg	Reduced CS in schizophrenic patients at spatial frequencies below 7 c/deg compared to controls
Cimmer et al. (2006)	44 schizophrenic patients, 20 controls	0.5 c/deg, 8 Hz	Reduced CS in schizophrenic patients compared to controls
Butler et al. (2009)	20 schizophrenic patients, 17 controls	0.5, 7, 21 c/deg	Reduced CS in schizophrenic patients at 0.5 c/deg compared to controls
Calderone et al. (2013)	15 schizophrenic patients, 15 controls	0.5, 4 c/deg	Reduced CS in schizophrenic patients compared to controls
Cadenhead et al. (2013)	53 schizophrenic patients, 22 schizotypal personality disorder, 53 controls.	1.22 c/deg, 8.33 Hz	Reduced CS in schizophrenic patients and those with schizotypal personality disorder compared to controls
Shoshina et al. (2014)	45 schizophrenic patients (25 treated with antipsychotics blocking serotonin and dopamine receptors, 20 treated with antipsychotics blocking dopamine receptor), 20 controls	0.4, 3.6, 17.9 c/deg	Reduced CS at 0.4 and 3.6 c/deg for both patient groups compared to controls Patients prescribed serotonin and dopamine receptor blockers had a larger deficit at 0.4 c/deg. Patients prescribed dopamine receptor blockers had a larger deficit at 3.6 and 17.9 c/deg
Samani et al. (2018)	24 schizophrenics 44 controls	0.5, 2, 8 c/deg	Reduced CS at 0.5 c/deg compared to controls.

0 Hz indicates that patterns were stationary

*CS* contrast sensitivity

Some of the differences between studies may be accounted for by the nature of contrast sensitivity testing, which is typically long and relatively labour-intensive. Instructions may sometimes be difficult for schizophrenic patients to understand. In addition, even for simple conventional tasks of contrast detection, response criteria used by patients to make decisions may differ from controls and between patients.

Another issue concerns the clinical characteristics of different groups of schizophrenics across different studies, both in terms of their symptoms and medication. This is likely to represent a particular problem in interpreting the findings of the present literature given that the numbers of schizophrenic patients included in studies are often relatively small. It is, therefore, difficult to make firm conclusions from individual studies. Indeed, in this context, it should be noted that schizophrenia has been suggested to be a heterogeneous condition; an umbrella term for a variety of overlapping conditions (Franzek and Beckmann 1998; Ban 2004). Further, the medications taken for schizophrenia might also contribute to variations amongst studies. For example, it has been suggested that typical antipsychotics, which tend to be antagonists at the dopamine D2 receptor, may improve contrast sensitivity in schizophrenic patients; whereas, atypical antipsychotics, where the mechanism of action is shifted towards blockade of the 5-HT2 receptor and less antagonism of the dopamine D2 receptor, may normalise it (Chen et al. 2003).

Schizotypy offers a means for understanding the aetiology of schizophrenia (Barrantes-Vidal et al. 2015). It also allows the determination of some characteristics of schizoaffective disorders, in our case, low-level visual disturbances such as reduced contrast sensitivity, in the absence of the potential confounds of medication. The term ‘schizotypy’ was coined by Meehl (1962) as a form of personality organisation commonly associated with an increased susceptibility to schizophrenia, although most schizotypes are not necessarily expected to develop psychosis (Barrantes-Vidal et al. 2015). In the ‘typical’ population, it can present as subtle, sub‐clinical manifestations of psychotic characteristics, often so subtle that it is undetectable (see Lenzenweger 2018 for a comprehensive review of schizotypy, schizotypic psychopathology and schizophrenia).

Although schizotypic traits often remain undetectable to others in everyday life, they are apparent from self-report measures. One of the most commonly used is The Oxford-Liverpool Inventory of Feelings and Experiences (O-Life) Questionnaire (Mason and Claridge 2006). It is made up of 104 items, which produce a score for each participant across 4 subscales: Unusual Experiences, Cognitive Disorganisation, Introverted Anhedonia and Impulsive Nonconformity. High scores on the unusual experiences subscale can manifest as perceptual aberrations, magical thinking, and hallucinations. In the context of psychosis, the unusual experiences subscale is phenomenologically related to positive symptoms. High scores on the cognitive disorganisation subscale reflect poor attention, concentration, and decision-making, and, in the context of psychosis, are phenomenologically related to thought disorder. High scores on the introverted anhedonia subscale reflect negative schizotypy, and manifest as lack of enjoyment, withdrawal and avoidance of intimacy. High scores on the impulsive nonconformity subscale reflect anti-social, and eccentric forms of behaviour, suggestive of a lack of self-control/inhibition and asocial behaviour.

In the present study, across 3 experiments, we sought to determine the existence of a relationship between schizotypy scores on the O-Life Questionnaire and spatial and temporal contrast sensitivity. The combination of spatial and temporal frequencies used was based on previous studies of reduced contrast sensitivity in schizophrenia and schizoaffective personality disorder. Experiment 1 investigated the effects of high and low schizotypy on spatial contrast sensitivity at spatial frequencies of 0.5, 2 and 8 c/deg. Experiment 2 investigated the effects of high and low schizotypy on contrast sensitivity for patterns (0.5 and 8 c/deg) drifting at low temporal frequencies (0.5 Hz). Experiment 3 investigated the effects of high and low schizotypy on contrast sensitivity patterns (0.5 and 8 c/deg) drifting at high temporal frequencies (8 Hz).

## Materials and methods

### Participants

An opportunity sample of 73 participants (68 females, 5 males), aged 18–26 years (mean 19.5 years; SD 1.8) took part in the study. Participants were undergraduate students in the School of Psychology at the University of Leicester. They had no history of ocular disease and reported that they were not taking any medications at the time of testing. Binocular corrected visual acuity at near and distance was in the normal range for all participants included in the study. Ethical approval was granted by the University of Leicester. All experimental methods adhered to the tenets of the Declaration of Helsinki. Informed consent was obtained before the study commenced. Upon admission to the study, participants completed the O-Life questionnaire.

### Apparatus and stimuli

Sinusoidal gratings subtended 6 degrees (horizontally and vertically) at a viewing distance of 69.5 cm and were generated using a Macintosh G4 and presented on a Sony Trinitron CRT monitor with an update rate of 75 Hz using the C programming language. The monitor was gamma-corrected using a spot photometer (LS-100, Konica Minolta) and look-up-tables (LUT). For precise control of luminance contrast, the number of intensity levels available was increased from 8 to 14 bits using a Bits ++ attenuator (Cambridge Research Systems). The mean luminance of the display was ~ 64 cd/m^2^ (min = 1.16; max = 127.7) and the monitor was the only light source. Total stimulus presentation duration was 853 ms and the luminance contrast of the sinusoidal waveform was smoothed on and off by half a cycle of a raised cosine lasting 170 ms. In a similar manner, the sinusoidal waveform was spatially windowed in the horizontal dimension according to a half cycle of a raised cosine function with a half-period of 1.2 deg. This was done to minimise the presence of spatial and temporal transients.

The Michelson contrast of the pattern could be varied according to the following equation:

1 $${\text{Luminance contrast }} = \left( {L_{ \hbox{max} } - \, L_{ \hbox{min} } } \right) \, / \, \left( {L_{ \hbox{max} } + \, L_{ \hbox{min} } } \right),$$

where *L*_max_ and *L*_min_ are the maximum and the minimum luminances of the grating, in the range 0–1.

### Procedure

Contrast threshold measurements were taken using a single-interval, forced-choice procedure. In Experiment 1, on each trial, participants were presented with a fixation cross, followed by the presentation of a stationary grating, upon which they were required to judge its orientation (vertical or horizontal). In Experiments 2 and 3, on each trial, participants were presented with a fixation cross, followed by the presentation of a moving pattern and required to judge its direction (left vs right). Before each experiment commenced, participants were allowed a short practice run. The luminance contrast of the test stimulus was varied from trial to trial according to a modified 3-down, 1-up staircase designed to converge on the contrast corresponding to 79.4% correct. At the beginning of each run of trials, the contrast of the test pattern was initially set to a suprathreshold level (typically ~ 6 dB above threshold) and the initial staircase step size was chosen to be half of this value. On subsequent reversals, the step size was halved and testing was terminated after a total of 16 reversals. Threshold estimates were taken as the mean of the last 4 reversals in each staircase. Each observer completed 2 staircases per condition and a mean was taken. The order of testing was randomised within each experiment.

### Data analysis

Median splits were performed for each O-Life subscale.^1^ This provided groups of high and low scorers on each subscale as follows: Unusual Experiences: *n* = 68 (35 high, 33 low); Cognitive Disorganisation: *n* = 65 (32 high, 33 low); Introverted Anhedonia: *n* = 63 (34 high, 29 low); Impulsive Non-conformity: *n* = 62 (33 high, 29 low). Contrast thresholds were converted to contrast sensitivity (1/contrast threshold) for graphical representations of data and statistical analyses. For Experiment 1, 3 (spatial frequency: 0.5, 2, 8 c/deg) by 2 (group: high, low), mixed, repeated measures analyses of variance were performed separately for each subscale. For Experiments 2 and 3, 2 (spatial frequency: 0.5, 8 c/deg) by 2 (group: high, low), mixed, repeated measures analyses of variance were performed separately for each subscale. Significant findings were investigated further using post hoc independent samples* t*-tests and regression analyses.

## Results

### Experiment 1: spatial contrast sensitivity

**Fig. 1:**
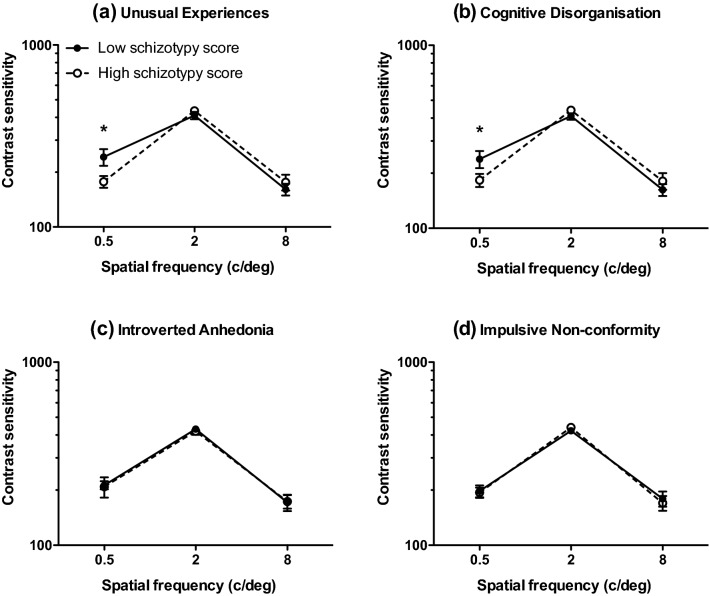
Mean contrast sensitivity at 0.5, 2 and 8 c/deg for those who scored ‘high’ (open symbols) and ‘low’ (closed symbols) on each O-Life subscale: **a** unusual experiences, **b** cognitive disorganisation, **c** introverted anhedonia and **d** impulsive non-conformity. Error bars are ± 1 SEM

**Fig. 2:**
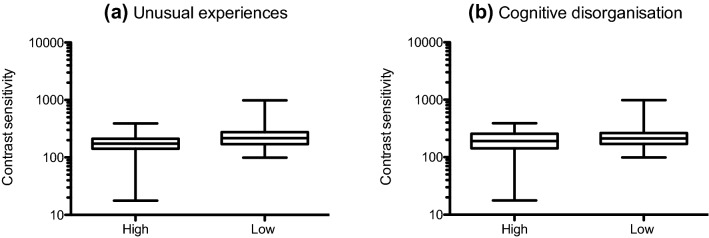
Box and whisker plots showing minimum, 1st quartile, median, 3rd quartile and maximum contrast sensitivity at 0.5 c/deg for those who scored ‘high’ or ‘low’ on the **a** unusual experiences and **b** cognitive disorganisation subscales of the O-Life Questionnaire

**Fig. 3:**
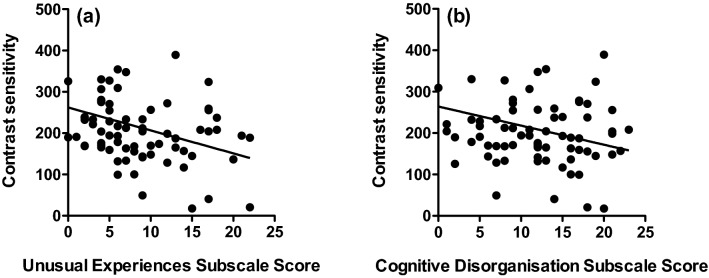
Individual scores on the **a** unusual experiences and **b** cognitive disorganisation subscales of the O-Life questionnaire against contrast sensitivity scores at 0.5 c/deg

Mean (± 95% confidence intervals) and median contrast thresholds at 0.5, 2 and 8 c/deg for those who scored ‘high’ and ‘low’ on each O-Life subscale are given in Table 2. Mean contrast sensitivity (1/contrast threshold) is shown in Fig. 1. 3 (spatial frequency: 0.5, 2, 8 c/deg) by 2 (group: high, low) analyses of variance performed separately for each subscale revealed significant main effects of spatial frequency on contrast sensitivity across all subscales as follows: Unusual Experiences: F(1.733,114.361) = 129.425; *p* < 0.001;η*p*^2^ = 0.662; Cognitive Disorganisation: F(1.682,105.980) = 122.753; *p* < 0.001; η*p*^2^ = 0.661; Introverted Anhedonia: F(1.656,101.023) = 109.160; *p* < 0.001; η*p*^2^ = 0.642; Impulsive Non-conformity: F(2120) = 242.195; *p* < 0.001; η*p*^2^ = 0.801. There were no main effects of group for any of the subscales. There were small but significant spatial frequency x group interactions for the subscales Unusual Experiences [F(1.733, 114.361) = 4.219; *p* = 0.011; η*p*^2^ = 0.060] and Cognitive Disorganisation [F(1.682,105.980) = 3.620; *p* = 0.016; η*p*^2^ = 0.054]. Post hoc, Bonferroni-corrected t-tests performed at each spatial frequency (0.5, 2, 8 c/deg) showed that this interaction reflected significantly lower contrast sensitivity at 0.5 c/deg in those who scored highly on the Unusual Experiences [*t* = − 2.278; df = 66; *p* = 0.013; *d* = 0.57] and Cognitive Disorganisation [*t* = − 230; df = 63; *p* = 0.034; *d* = 0.47] subscales. To provide a better representation of the distribution of contrast sensitivity scores for high and low schizotypes, for conditions on which significant differences emerged (unusual experiences and cognitive disorganisation subscales at 0.5 c/deg), box and whisker plots are provided in Fig. 2. It is evident that, although schizotypy had a significant effect under these conditions, there was considerable overlap between groups. Further, regression analyses confirmed that individual scores on each subscale significantly predicted contrast sensitivity at low spatial frequencies (Unusual Experiences: *R*^2^ = 0.047, *F*(1,71) = 3.56 *p* = 0.031; Cognitive Disorganisation: *R*^2^ = 0.053, *F*(1,71) = 3.95 *p* = 0.026), shown in Fig. 3.

**Table 2:** Mean (with lower (c) and upper ( + ) 95% confidence intervals) and median contrast thresholds (in the range 0–1) at 0.5, 2 and 8 c/deg for high and low schizotypes on each O-Life subscale

Spatial frequency	Subscale	Score	Mean (± 95% CIs)	Median
0.5 c/deg	Unusual experiences	High	0.0092 ( − CI: 0.0052; + CI: 0.0132)	0.0057
		Low	0.0049 ( − CI: 0.0043; + CI: 0.0055)	0.0046
	Cognitive disorganisation	High	0.0090 ( − CI: 0.0049; + CI:0.0132)	0.0052
		Low	0.0049 ( − CI: 0.0043; + CI:0.0056)	0.0047
	Introverted anhedonia	High	0.0075 ( − CI: 0.0043; + CI: 0.0107)	0.0053
		Low	0.0050 ( − CI: 0.0045; + CI: 0.0055)	0.0049
	Impulsive nonconformity	High	0.0074 ( − CI: 0.0044; + CI: 0.0105)	0.0052
		Low	0.0071 ( − CI: 0.00355; + CI: 0.0108)	0.0053
2 c/deg	Unusual experiences	High	0.0024 ( − CI: 0.0022; + CI: 0.0028)	0.0023
		Low	0.0027 ( − CI: 0.0023; + CI: 0.0032)	0.0024
	Cognitive disorganisation	High	0.0024 ( − CI: 0.0022; + CI:0.0027)	0.0022
		Low	0.0027 ( − CI: 0.0023; + CI:0.0032)	0.0024
	Introverted anhedonia	High	0.0026 ( − CI: 0.0022; + CI: 0.0031)	0.0024
		Low	0.0024 ( − CI: 0.0022; + CI: 0.0026)	0.0024
	Impulsive nonconformity	High	0.0024 ( − CI: 0.0022; + CI:0.0026)	0.0022
		Low	0.0025 ( − CI: 0.0023; + CI: 0.0028)	0.0024
8 c/deg	Unusual experiences	High	0.0077 ( − CI: 0.0062; + CI: 0.0093)	0.0065
		Low	0.0098 ( − CI: 0.0043; + CI: 0.0153)	0.0058
	Cognitive disorganisation	High	0.0079 ( − CI: 0.0057; + CI:0.0102)	0.0054
		Low	0.0075 ( − CI: 0.0062; + CI:0.0089)	0.0058
	Introverted anhedonia	High	0.0072 ( − CI: 0.0060; + CI: 0.0084)	0.0058
		Low	0.0109 ( − CI: 0.0043; + CI: 0.0174)	0.0058
	Impulsive nonconformity	High	0.0098 ( − CI: 0.0043; + CI: 0.0152)	0.0065
		Low	0.0079 ( − CI: 0.0054; + CI: 0.0106)	0.0056

### Experiment 2: spatiotemporal contrast sensitivity at low temporal frequencies

**Fig. 4:**
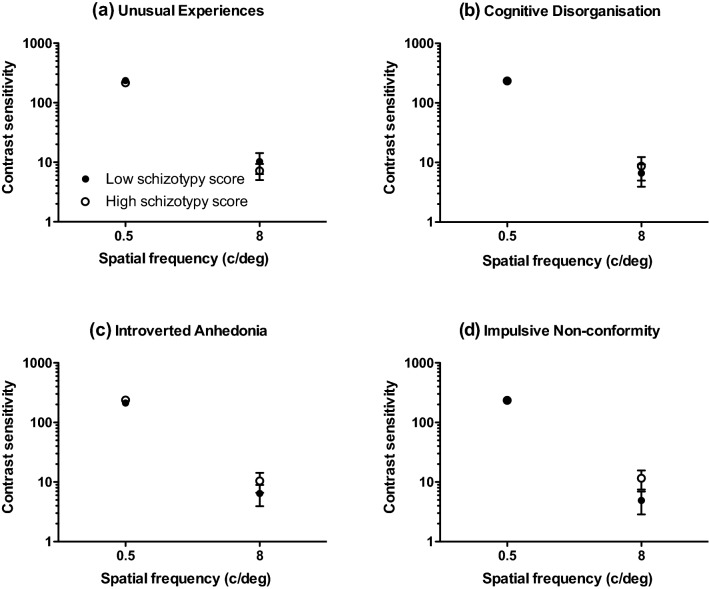
Mean contrast sensitivity for 0.5 and 8 c/deg patterns drifting at 0.5 Hz for those who scored ‘high’ and ‘low’ on each O-Life subscale: **a** unusual experiences, **b** cognitive disorganisation, **c** introverted anhedonia and **d** impulsive non-conformity. Error bars are ± 1 S.E.M

Table 3 gives mean (± 95% confidence intervals) and median contrast thresholds for 0.5 and 8 c/deg patterns drifting at 0.5 Hz for those who scored ‘high’ and ‘low’ on each O-Life subscale. Mean contrast sensitivity (1/contrast threshold) is shown in Fig. 4. A 2 (spatial frequency: 0.5, 8 c/deg) by 2 (group: high, low) analysis of variance performed separately for each subscale revealed significant main effects of spatial frequency on contrast sensitivity across all subscales as follows: Unusual Experiences: *F*(1,66) = 207.385; *p* < 0.001; η*p*^2^ = 0.759; Cognitive Disorganisation: *F*(1,63) = 214.607; *p* < 0.001; η*p*^2^ = 0.773; Introverted Anhedonia: *F*(1,61) = 203.896; *p* < 0.001; η*p*^2^ = 0.770; Impulsive Non-conformity: *F*(1,60) = 195.132; *p* < 0.001; η*p*^2^ = 0.765. There were no effects of group nor were there any spatial frequency* x* group interactions.

**Table 3:** Mean (with lower ( − ) and upper ( + ) 95% confidence intervals) and median contrast thresholds (in the range 0-1) for 0.5 and 8 c/deg patterns drifting at 0.5 Hz for each O-Life subscale

Spatial frequency	Subscale	Schizotypy group	Mean (± 95% CIs)	Median
0.5 c/deg	Unusual experiences	High	0.0064 ( − CI: 0.0047; + CI: 0.0080)	0.0047
		Low	0.0064 ( − CI: 0.0036; + CI: 0.0091)	0.0047
	Cognitive disorganisation	High	0.0057 ( − CI: 0.0040; + CI:0.0073)	0.0046
		Low	0.0066 ( − CI: 0.0038; + CI: 0.0094)	0.0048
	Introverted anhedonia	High	0.0064 ( − CI: 0.0037; + CI: 0.0091)	0.0047
		Low	0.0059 ( − CI: 0.0044; + CI: 0.0073)	0.0047
	Impulsive nonconformity	High	0.0056 ( − CI: 0.0041; + CI: 0.0070)	0.0047
		Low	0.0058 ( − CI: 0.0042; + CI: 0.0074)	0.0046
8 c/deg	Unusual experiences	High	0.6604 ( − CI: 0.5271; + CI: 0.7937)	0.8933
		Low	0.6561 ( − CI: 0.5182; + CI: 0.7940)	0.8242
	Cognitive disorganisation	High	0.6138 ( − CI: 0.4780; + CI: 0.7495)	0.7145
		Low	0.7565 ( − CI: 0.6299; + CI: 0.8830)	0.9663
	Introverted anhedonia	High	0.6400 ( − CI: 0.4975; + CI: 0.7825)	0.8612
		Low	0.6277 ( − CI: 0.4827; + CI: 0.7727)	0.7732
	Impulsive nonconformity	High	0.6366 ( − CI: 0.4895; + CI: 0.7838)	0.8933
		Low	0.6904 ( − CI: 0.5564; + CI: 0.8244)	0.8242

### Experiment 3: spatiotemporal contrast sensitivity at high temporal frequencies

**Fig. 5:**
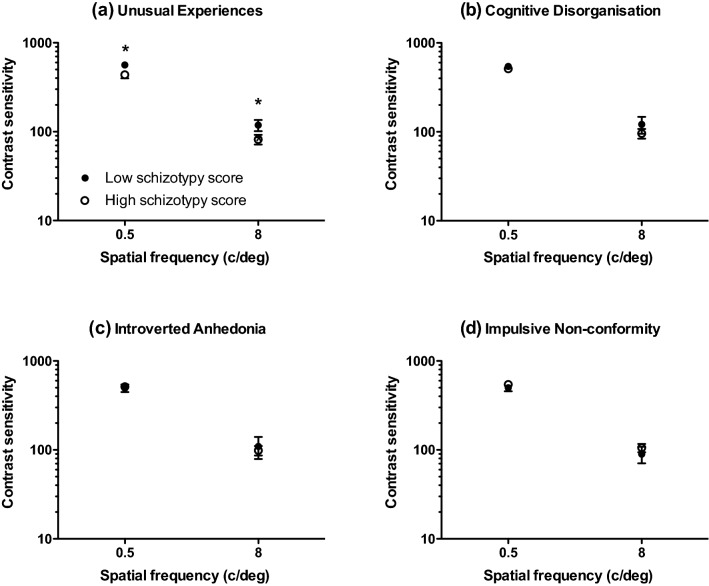
Mean contrast sensitivity for 0.5 and 8 c/deg patterns drifting at 8 Hz for those who scored ‘high’ and ‘low’ on each O-Life subscale: **a** unusual experiences, **b** cognitive disorganisation, **c** introverted anhedonia and **d** impulsive non-conformity. Error bars are ± 1 S.E.M

**Fig. 6:**
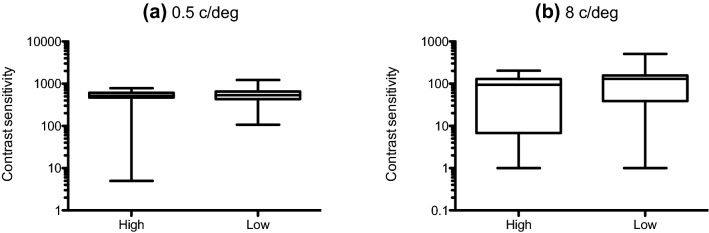
Box and whisker plots showing minimum, 1st quartile, median, 3rd quartile and maximum contrast sensitivity at **a** 0.5 c/deg and **b** 8 c/deg for patterns drfiting at 8 Hz in those who scored ‘high’ or ‘low’ on the unusual experiences subscale of the O-Life questionnaire

Table 4 gives mean (± 95% confidence intervals) and median contrast thresholds for 0.5 and 8 c/deg patterns drifting at 8 Hz for those who scored ‘high’ and ‘low’ on each O-Life subscale. Mean contrast sensitivity (1/contrast threshold) is shown in Fig. 5. A 2 (spatial frequency: 0.5, 8 c/deg) by 2 (group: high, low) analysis of variance performed separately for each subscale revealed significant main effects of spatial frequency on contrast sensitivity across all subscales as follows: Unusual Experiences: *F*(1,66) = 203.00; *p* < 0.001;η*p*^2^ = 0.755; Cognitive Disorganisation: *F*(1,63) = 202.876; *p* < 0.001;η*p*^2^ = 0.763; Introverted Anhedonia: *F*(1,61) = 188.414; *p* < 0.001;η*p*2 = 0.755; Impulsive Non-conformity: *F*(1,60) = 183.508; *p* < 0.001;η*p*^2^ = 0.754. There was a small but significant, main effect of group for the subscale ‘Unusual Experiences’ [*F*(1,66) = 3.278; *p* = 0.037;η*p*^2^ = 0.047], in that those categorised as ‘high’ schizotypes exhibited worse contrast sensitivity. There was no spatial frequency by group interaction, indicating that contrast sensitivity was significantly impaired at both spatial frequencies. The distributions of contrast sensitivity scores for high and low schizotypes on the unusual experiences O-Life subscale are shown in Fig. 6.

**Table 4:** Mean (with lower ( − ) and upper ( + ) 95% confidence intervals) and median contrast thresholds (in the range 0–1) for 0.5 and 8 c/deg patterns drifting at 8 Hz for each O-Life subscale

Spatial frequency	Subscale	Schizotypy group	Mean (± 95% CIs)	Median
0.5 c/deg	Unusual experiences	High	0.013 ( − CI: 0.0004; +CI: 0.0256)	0.0019
		Low	0.0026 ( − CI: 0.0018; +CI: 0.0032)	0.0019
	Cognitive disorganisation	High	0.0069 ( − CI: 0.0006; + CI:0.0133)	0.0018
		Low	0.0027 ( − CI: 0.0017; + CI: 0.0036)	0.0018
	Introverted anhedonia	High	0.0029 ( − CI: 0.0015; + CI: 0.0044)	0.0018
		Low	0.0128 ( − CI: 0.0022; + CI: 0.0278)	0.0019
	Impulsive nonconformity	High	0.0099 ( − CI: 0.0026; + CI: 0.0224)	0.0018
		Low	0.0064 ( − CI: 0.0002; + CI: 0.0126)	0.0019
8 c/deg	Unusual experiences	High	0.1814 ( − CI: 0.0648; + CI: 0.2979)	0.0106
		Low	0.1202 ( − CI: 0.0231; + CI: 0.2174)	0.0077
	Cognitive disorganisation	High	0.1395 ( − CI: 0.0297; + CI: 0.2493)	0.0095
		Low	0.1131 ( − CI: 0.0217; + CI: 0.2044)	0.0098
	Introverted anhedonia	High	0.1144 ( − CI: 0.0250; + CI: 0.2038)	0.0092
		Low	0.1935 ( − CI: 0.0604; + CI: 0.3266)	0.0135
	Impulsive nonconformity	High	0.1072 ( − CI: 0.0165; + CI: 0.1979)	0.0087
		Low	0.1677 ( − CI: 0.0469; + CI: 0.2885)	0.0134

Regression analyses did not reveal any significant associations between individual scores on the Unusual Experiences subscale and contrast sensitivity at these spatiotemporal frequencies.

## Discussion

The results of the present study revealed schizotypy-related differences in spatial and spatiotemporal contrast sensitivity. Spatial contrast sensitivity was significantly lower in high, compared to low schizotypes at low spatial frequencies (0.5 c/deg) but not intermediate (2 c/deg) or higher (8 c/deg) spatial frequencies. These differences were subscale specific, only being evident on the Unusual Experiences and Cognitive Disorganisation subscales. For moving stimuli, individuals with high scores on the Unusual Experiences subscale exhibited lower spatiotemporal contrast sensitivity at higher (8 Hz), but not lower (0.5 Hz) temporal frequencies. Scores on the introverted anhedonia and impulsive nonconformity subscales had no effect on contrast sensitivity across any spatial and/or temporal frequencies.

Deficits were most pronounced for spatial contrast sensitivity (i.e., sensitivity to the contrast of stationary patterns). These results are in keeping with studies in schizophrenia where reduced contrast sensitivity is most commonly found for stationary patterns. In terms of the spatial frequency profile of reduced contrast sensitivity, these were only evident in high schizotypes at low spatial frequencies. In schizophrenics, reduced contrast sensitivity has been shown to selectively affect low to intermediate spatial frequencies (Butler et al. 2005, 2009; Shoshina et al. 2014; Samani et al. 2018). However, other studies have found more non-selective spatial contrast sensitivity across a range of spatial frequencies (Slaghuis 1998; Keri et al. 2002; Chen et al. 2003), whereas others find no deficit (Chen et al. 1999). For moving patterns, we found selectively reduced contrast sensitivity at high (8 Hz), but not low (0.5 Hz) temporal frequencies, irrespective of the spatial frequencies (0.5, 8 c/deg) we used. In schizophrenia, reduced contrast sensitivity for moving test patterns has been shown in some (Keri et al. 2002; Chen et al. 2003; Slaghuis 2004; Cimmer et al. 2006; Cadenhead et al. 2013), but not all (Chen et al. 1999) schizophrenic groups across temporal frequencies ranging from 0.5 to 16 Hz. Improved contrast sensitivity has also been documented in schizophrenia under some conditions (Chen et al. 2003). Our findings at high temporal frequencies might also reflect increased internal noise (decreased signal–noise ratios) in those who exhibited high schizotypy scores. In the context of schizophrenia, a recent study (Chen et al. 2014) using random dot kinematograms has shown that schizophrenics exhibited significantly poorer speed discrimination across a range of speeds (5.25–13.0 deg/s) and their performance was more susceptible to the addition of noise compared to controls.

One finding of note is that, even for spatial contrast sensitivity, deficits were only apparent in individuals who scored highly on the unusual experiences and cognitive disorganisation subscales of the O-Life questionnaire. For moving patterns, deficits at high temporal frequencies were only apparent for those who scored highly on the unusual experiences subscales. That reduced contrast sensitivity in high schizotypes was subscale specific may be accounted for by the nature of the schizotypic characteristics comprised within each subscale. High scores on the unusual experiences subscale manifest as perceptual aberrations, magical thinking, and hallucinations, commonly associated with positive symptoms of schizophrenia. The characteristics comprised in this subscale are considerably less subtle than those represented in other subscales (e.g., the lack of enjoyment and withdrawal and avoidance of intimacy encapsulated by the introverted anhedonia subscale, and the components of anti-social and eccentric behaviour in the impulsive nonconformity subscale). That reduced spatial contrast sensitivity was evident in those who scored highly on the cognitive disorganisation scales, represented by poor attention, concentration, and decision-making, are in keeping with other studies that consistently document difficulties with attention and cognition in both high schizotypes and schizophrenics (Luck and Gold 2008; Ettinger et al. 2015).

One possible caveat of the findings presented here is that our sample was restricted to young adults, the majority of whom were female. As such, it may be that our findings would not necessarily generalise beyond a sample of this type. For example, using the Schizotypal Personality Questionnaire (SPQ), Bora and Baysan Arabaci (2009) have presented evidence that some schizotypal personality traits may be most prevalent in younger adults, becoming less pronounced with age. Of particular relevance to the present study is the finding that, compared to older age groups, younger participants exhibited higher scores on the unusual perceptual experiences subscale of the SPQ. Some gender differences in schizotypal characteristics have also been reported. Where male participants tend to exhibit higher scores than female participants on disorganised and negative symptom‐like aspects of schizotypy (Mata et al. 2005; Bora and Baysan Arabaci 2009), female participants tend to exhibit higher scores on scale items related to social anxiety, magical thinking, paranoia and odd beliefs (Mata et al. 2005; Fonseca-Pedrero et al. 2008; Bora and Baysan Arabaci 2009).

In conclusion, we have shown reduced contrast sensitivity in high schizotypy for stationary and moving test patterns. Such deficits were most pronounced for stationary test patterns at low spatial frequencies in schizotypic individuals with a propensity towards unusual experiences and cognitive disorganisation. We also found reduced contrast sensitivity at high temporal frequencies (8 Hz) in those with a propensity towards unusual experiences. That reduced contrast sensitivity is evident in schizotypy is commensurate with the majority of findings in schizophrenics for whom reduced contrast sensitivity has been documented (although see Table 1 for variations between studies). In a broader sense, the findings presented here lend weight to the notion that schizotypy provides a useful construct for understanding the expression of psychopathology in schizophrenia.

## Footnotes

1 There are a number of ways in which we could have split our data into ‘high’ and ‘low’ schizotypy. For example, if we had only included participants with very high and very low values, any differences we found may have been more pronounced and/or stronger interactions may have emerged between some conditions. However, the purpose of our study was to determine whether subtle differences in schizotypal personality traits are reflected in contrast sensitivity scores in a typical/general population. As such, we performed a standard median split on the data, i.e., any scores above the median were assigned to ‘high’ and any scores below the median were assigned to ‘low’.
